# Brittle Materials in Mechanical Extremes

**DOI:** 10.3390/ma13204610

**Published:** 2020-10-16

**Authors:** Giovanni Bruno

**Affiliations:** BAM, Bundesanstalt für Materialforschung und prüfung, Unter den Eichen, 87, 12205 Berlin, Germany; Giovanni.bruno@bam.de; Tel.: +49-30-8104-1850

**Keywords:** ceramics, concrete, asphalt, mechanical properties, microstructure, microcracking, strength

## Abstract

The goal of the Special Issue “Brittle Materials in Mechanical Extremes” was to spark a discussion of the analogies and the differences between different brittle materials, such as, for instance, ceramics and concrete. Indeed, the contributions to the Issue spanned from construction materials (asphalt and concrete) to structural ceramics, reaching as far as ice. The data shown in the issue were obtained by advanced microstructural techniques (microscopy, 3D imaging, etc.) and linked to mechanical properties (and their changes as a function of aging, composition, etc.). The description of the mechanical behavior of brittle materials under operational loads, for instance, concrete and ceramics under very high temperatures, offered an unconventional viewpoint on the behavior of brittle materials. This is not at all exhaustive, but a way to pave the road for intriguing and enriching comparisons.

As a premise, it must be said that brittle materials are such an enormous category of materials that it would be impossible to disclose and even comment on their mechanical properties in a short editorial. The following text is therefore limited to the contents of the Special Issue “Brittle Materials in Mechanical Extremes”, and to some of the works that the editor has conducted, leading to the initiation of the Special Issue.

Brittle materials include a wide range of material classes: From polymers to metals, through to classic glass, ceramics, and composites. They all share a supposed linear elastic behavior but are often found to display non-linear stress–strain relationships or high temperature dilation (or other properties such as thermal conductivity). In this Special Issue, contributions describing and explaining this intriguing behavior, whether due to microcracking, interaction among constituent phases, or micro-structural features (such as pores), were collated. Advanced characterization techniques, challenging numerical and analytical models, unconventional experiments, and/or the analysis of existing field data were reported and should spark the debate about the origin of the mechanical behavior of brittle materials under various and unconventional mechanical, weathering, and thermal loads.

The Special Issue allows (through the large amount of experimental data provided by the contributing authors) a discussion of the analogies and the differences between different materials, such as, e.g., ceramics and concrete. Data have been corroborated by advanced microstructural studies (microscopy, 3D imaging, etc.), sometimes leading to the identification of the microstructure–property relationships through appropriate models.

A few works have demonstrated the peculiar behavior of brittle materials, such as concrete (see the book of Torrenti et al. [1]), composite materials (see e.g., [2]), and ceramics (see e.g., [3,4]), especially if they possess special features such as pores or hard phases, or undergo microcracking. Microcracks, together with porosity, impart to such materials an exceedingly large strain tolerance (strain at failure), where the specimen is able to carry some (small) load even when it is fully fractured (thereby displaying residual strength [5]), excellent corrosion resistance [6], or extremely high thermal shock resistance [7]. In this sense, such materials behave similarly to metamaterials, where the meso-structure (at scales above the grain size) is as important as the material composition of the micro-structure (at scale below the grain size). Simply thinking of microcracked glass or ceramics, possessing negative macroscopic thermal expansion [8], exemplifies such astonishing behavior.

Several techniques have been used in the literature (and were used in this Special Issue) to characterize this behavior: instrumented indentation (see, for instance, the book of Buljak [9], or the contribution [10] to this Special Issue) is particularly suitable to monitor the variation of the Young’s modulus as a function of the load, thereby allowing insights into “mechanical microcracking” (see [11] for its definition). Moreover, indentation allows for the use of inverse methods to extract the (non-linear) constitutive behavior of materials [12]. If the results obtained by the application of inverse methods are verified by independent experiments (as done for example in Reference [13]), the fitting parameters acquire the meaning of materials’ properties. An example of this approach is also given in the Special Issue [14]. In general, modeling is now a requirement for good experimental data to be fully exploited. Coupling even simple models to data on the mechanical behavior extends the validity of such data to a more universal level. This is what was practiced in this Special Issue, whether with creep models for concrete or damage models for asphalt [15], thereby setting a standard for good scientific practice. 

From an experimental point of view, this Special Issue demonstrated that inventive is needed to solve the problem of the characterization of the relevant quantities in brittle in materials. Special set-ups [16] or unconventional test methods [17] were utilized to disclose the damage behavior of such materials. In order to characterize the microstructure, the use of 3D imaging techniques (usually X-ray based) is becoming increasingly standardized in materials science to disclose meso-structures (pores, cracks, inhomogeneities), while the microstructural characterization is still classically based on optical microscopy (OM) and scanning electron microscopy (SEM) pictures. Composite materials, porous ceramics and concrete all carry meso-structural features (again, with sizes above the crystal grain) that can be well imaged by laboratory computed tomography. Apart from carrying an augmented information content, 3D data such as those from X-ray computed tomography, optical tomography or 3D infra-red thermography, allow the use of finite element simulations based on experimentally determined microstructures, or the extraction of quantitative data (e.g., fiber orientation distribution in concrete [18]) at the scale of a representative volume element (RVE). On top of that, in this Special Issue, the strength of a relatively novel technique, X-ray refraction radiography (see [19] for an introduction to it) was successfully used to quantify damage in thermally cycled refractory ceramics [20].

Finally, the description of the mechanical behavior of brittle materials under operational (sometimes unconventional) loads, such as mechanical and temperature cycling, electric fields, corrosion environments, while already underway in the literature, represents the natural extension of this Special Issue. 
